# Co-Pyrolysis of Cotton Stalks and Low-Density Polyethylene to Synthesize Biochar and Its Application in Pb(II) Removal

**DOI:** 10.3390/molecules27154868

**Published:** 2022-07-29

**Authors:** Xiaowei Yuan, Xuejun Zhang, Huijie Lv, Yonggang Xu, Tianxia Bai

**Affiliations:** 1 School of Mechanical and Electrical Engineering, Xinjiang Agricultural University, Urumqi 830052, China; ywei5199@163.com; 2 Bayingol Vocational and Technical College, Korla 841000, China; lvhuijie2003@126.com; 3 Jiangsu Key Laboratory for Eco-Agricultural Biotechnology around Hongze Lake/Collaborative Innovation Center of Regional Modern Agriculture & Environmental Protection, Huaiyin Normal University, Huai’an 223300, China; baitianxia2008@163.com

**Keywords:** cotton stalk, polyethylene, biochar, pyrolysis, adsorption

## Abstract

It is inevitable that reclaimed cotton stalks will contain a certain amount of plastic film due to the wide application of plastic mulching during the process of cotton cultivation, and this makes it inappropriate to return it to the field or for it to be processed into silage. In this study, biochars were prepared by the co-pyrolysis of cotton stalk with low-density polyethylene (LDPE) in the proportions of 1:0, 3:1, 2:1, and 1:1 (*w*/*w*) at 400 °C, 450 °C, and 500 °C and maintaining them for 1 h. The effects of the co-pyrolysis of cotton stalk with LDPE on the properties of biochars (e.g., pH, yield, elemental analysis, specific surface area, etc.) and the Pb(II) removal capacity were analyzed. Co-pyrolysis cotton stalks with LDPE could delay the decomposition of LDPE but could promote the decomposition of cotton stalk. At 400 °C and 450 °C, the addition of LDPE decreased the H/C ratio, while no significant difference was found between the pristine biochar and the blended biochar pyrolyzed at 500 °C. An FTIR analysis indicated that the surface functional groups of biochar were not affected by the addition of LDPE, except for CH_3_ and CH_2_. The results of the SEM showed that LDPE could cover the surface of biochar when pyrolyzed at 400 °C, while many macropores were found in the blended biochar that was pyrolyzed at 450 °C and 500 °C, thus increasing its surface area. The blended biochar that was pyrolyzed at 500 °C was more effective in the removal of Pb(II) than the cotton-stalk-derived biochar, which was dominated by monolayer adsorption with a maximum adsorption capacity of approximately 200 mg·g^−1^. These results suggested that the co-pyrolysis of cotton stalks and LDPE may be used to produce biochar, which is a cost-effective adsorbent for heavy metal removal from aqueous solutions.

## 1. Introduction

In China, cotton (*Gossypium hirsutum* L.) is one of the most important agricultural crops and many tonnages of cotton stalk waste are generated from the agricultural production of cotton. At present, more than 20 million tons (dry weight) of cotton stalks are produced annually [1]. As cotton stalks are not favorable feed for livestock, the majority of it is directly incinerated in the field, resulting in serious environmental pollution [2]. Furthermore, a considerable portion of cotton stalks are crushed and mulched into soil, which has negative impacts on cultivation [3]. Pyrolysis is one of the promising technologies employed for biomass, and it can convert biomass into high-value-added products, such as biogas, bio-oil and biochar. Cotton stalks-derived biochar showed the greatest potential as a soil amendment to improve the fertility of soils as well as to sequester carbon [4], and for the removal of contaminants from water [5].

Plastic mulching has been widely adopted for industrial crop production, including for cotton [3]. Generally, the plastic film can be retained during the whole growth season, especially in arid and special early-maturing cotton areas to improve soil temperature and decrease moisture evaporation [6]. More than 2 million tons of plastic film residues, accounting for 20–25% of the total plastic film applied, remains in the soil in China [7]. Therefore, it is inevitable that the reclaimed cotton stalks contains a certain amount of plastic film, and this has also made it inappropriate to return it to the field or to be processed into silage. Therefore, it is extremely urgent to develop an efficient way to treat such waste mixtures.

Co-pyrolysis has been proven be a promising method for treating a high number of different wastes simultaneously, due to its reduced operation time and energy consumption [8,9]. The co-pyrolysis of biomass and different types of plastics has been frequently studied; it should be noted that most studies on this topic focused on the interactions on thermal behavior and kinetics analysis [10,11,12]. Furthermore, some studies have demonstrated that co-pyrolysis could generate a positive synergistic interaction between improving the yield and the properties of production when compared to conventional pyrolysis technology [13,14,15]. Due to the use of different species and mix ratios of feedstock and pyrolysis conditions, a variety of conclusions on the characteristics of pyrolytic production have been acquired. For example, Rathnayake et al. (2021) [14] found that the presence of plastic in feedstock mostly had negative effects on the C and H content of biochars derived from the spent strawberry growing medium and a mostly positive effect on those of the bean crop residue-derived biochars [14]. During pyrolysis, cellulose, hemicellulose, and lignin behave differently, and also some extent of interaction may occur, which increases the complexity of the overall process. Cotton stalks have a relatively higher cellulose content compared to other biomass [16]. However, the physicochemical properties of the biochar produced by the co-pyrolysis of cotton stalks and plastic have not been researched.

Biochar has been intensively used as a sorbent for contaminants [17]. However, in recent research, biochar was found to have a limited ability to adsorb high levels of contaminants. The adsorption capacity of biochar is directly related to its physicochemical properties, such as surface area, functional groups and cation-exchange capacity [18]. As stated above, the properties of biochar could be improved via the co-pyrolysis of biomass and plastic. Therefore, some studies have also found that the co-pyrolysis of biomass and plastic may be favorable for enhancing the adsorption of contaminants [19,20]. However, to the best of our knowledge, no comparative studies have been conducted and reported on the application of biochar derived from cotton stalks and plastic to heavy metal adsorption. 

In this work, we selected LDPE as feedstock because it is the most commonly used plastic film type. The main objective of this study was to investigate (1) the effect of different LDPE addition ratios on the pyrolysis behavior of cotton stalks, (2) its effects on biochar yield and the properties under the condition of different pyrolysis temperatures, and (3) the performance of biochar as a sorbent for Pb(II) in water and the factors affecting this. Here, the possible mechanism for Pb(II) adsorption is also discussed.

## 2. Materials and Methods

### 2.1. Feedstock Collection and Biochar Production

Corn stalks were obtained from a farmland in Bayingolin Mongolian Autonomous Prefecture, Xinjiang, China. Obtained corn stalks were first dried in an oven for 12 h at 105 °C, and then the samples were ground and sieved using a 60 mesh. Powdered LDPE (60 mesh) was purchased from Zhonglian plastics company. The pretreated cotton stalks and LDPE were fully mixed using an agitator in the mass ratios of 1:0, 3:1, 2:1, and 1:1, which are hereafter referred to as C1P0X, C3P1X, C2P1X, and C1P1X, for produced biochar, respectively, where X indicates the pyrolysis temperature. Then, these samples were placed into a porcelain crucible and thermally treated in a vacuum tube furnace under a 300 mL min^−1^ N_2_ flow condition, as shown in Figure S1. The furnace was heated to a designated temperature (400 °C, 450 °C, or 500 °C) at a heating rate of 10 °C min^−1^ and this was maintained for 1h. The produced biochars were packed in sealed plastic bags for analysis.

### 2.2. Thermogarvimetric Analysis

Thermogravimetric (TG) analysis of the samples were conducted in a TG analyzer (STA449F3, NETZSCH, Selb, Germany). In detail, about 10 mg of the sample was placed in an Al_2_O_3_ crucible and heated from 50 °C to 800 °C at a heating rate of 10 °C min^−1^ in an N_2_ atmosphere (99.999% purity at a flow rate of 50 mL min^−1^).

### 2.3. The Analysis of Feedstock and Biochar Properties

The pH values of biochars (sample/deionized water, 1:20, *w*/*v*) were measured using a pH meter (PHS-3C, Sanxin, Shenzhen, China). The ash content was measured according to the mass reduction after heating under 650 °C for 3 h in a muffle furnace. The carbon (C), hydrogen (H), and nitrogen (N) contents were determined using an automatic elemental analyzer (Vario EL cube, Elementar, Langenselbold, Germany). Oxygen content was calculated based on the difference on an ash-free basis. 

The surface area and pore properties of samples were determined using the results of N_2_ sorption tests conducted at 77 K using the Brunaumer–Emmett–Teller (BET) method (ASAP 2020, Micromeritics, Norcross, GA, USA). The surface structure of these samples was analyzed by employing Scanning Electron Microscopy (SEM, FEI Inspect S50, Hillsboro, OR, USA). Fourier Transform Infrared (FTIR) spectra of the above samples were recorded using a Nicolet iS50 spectrometer (Thermo Fisher Scientific, Waltham, MA, USA) in the region of 400–4000 cm^−1^.

### 2.4. Pb(II) Adsorption Experiment

The batch adsorption experiments of Pb(II) on biochars were carried out to determine the effects of various parameters (mixing ratios, pyrolysis temperature, pH and contact time). The isotherm adsorption experiments were conducted in 50 mL polypropylene centrifuge tubes containing 0.10 g biochar and 20 mL solution of varied Pb(II) concentrations (50, 100, 200, 400, and 800 mg L^−1^) at pH= 5.0. The kinetic adsorption experiments were performed for different contact times (0.5, 1, 2, 4, 8, 24, and 48 h) at an initial Pb(II) concentration of 200 mg L^−1^ at pH 5.0. To examine the effect of pH on adsorption, the initial pH values of the mixtures were adjusted to 2.0, 3.0, 4.0, 5.0, 6.0, and 7.0 ± 0.1 using 0.1 M or 0.01 M HNO_3_ and NaOH. An Inductively Coupled Plasma Optical Emission Spectrometer (Optima 2000DV, Perkin-Elmer, Waltham, MA, USA) was used to analyze the Pb(II) concentrations.

The adsorption capacity (*q_e_*, mg g^−1^) and removal efficiency (%) are calculated as:

(1) $$q_{e}=\frac{V\times \left(C_{i}-C_{e}\right)}{M}$$

(2) $$Removal\;efficiency\;\left(\%\right)=\frac{\left(C_{i}-C_{e}\right)}{C_{i}\times 100\%}$$

where *C_i_* and *C_e_* are the initial and final metal concentrations of the adsorption process (mg L^−1^), respectively; *V* is the solution volume (L); *M* is the biochar mass (g).

The analysis of adsorption (kinetic and isotherm) is provided in the Supplementary Material (Text S1). All parameters were calculated via nonlinear regression analysis using Origin 8.0 (Origin Lab Corporation, Northampton, MA, USA).

## 3. Results and Discussion

### 3.1. Thermogravimetric Analysis

**Figure 1:**
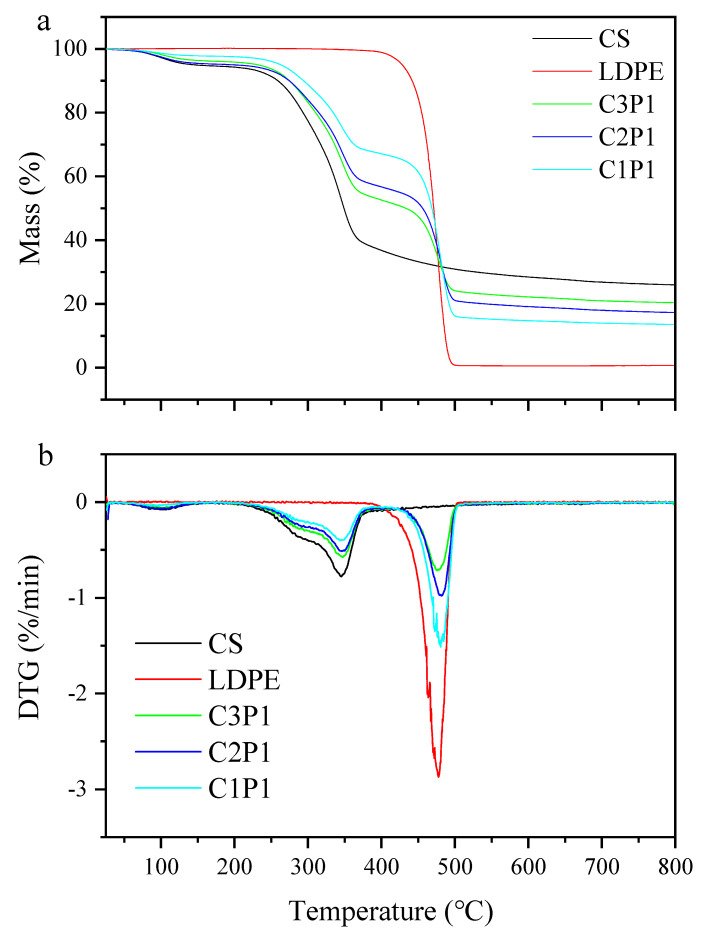
TG (**a**) and DTG (**b**) curves of cotton stalks, LDPE, and blends.

The TG and DTG curves of the cotton stalks with and without the LDPE addition are shown in Figure 1. Approximately 26 wt% of the cotton stalks remained after pyrolysis at 800 °C, as depicted in Figure 1a. It can be clearly seen that the mass loss of cotton stalks can be divided into two main stages. The first stage (50–190 °C) only has a minor mass loss caused by the release of H_2_O. The second stage occurred in the range of 210–400 °C, which corresponded to the decomposition of lignocellulose [15,21]. Only one mass loss of LDPE occurred within the temperature range between 400 and 500 °C, and LDPE was degraded completely at approximately 500 °C, which was consistent with a previous study [22]. 

When LDPE was mixed with cotton stalks, the degradation curve could be divided into two stages. The first stage (210–400 °C) was mainly associated with the decomposition of cotton stalks, which occurred because the LDPE did not begin to degrade before the temperatures reached 400 °C. It was noteworthy that the first peak temperatures of C3P1 (347.2 °C), C2P1 (345.7 °C), and C1P1 (344.3 °C) were lower than those of cotton stalks without LDPE (348.8 °C), suggesting that the addition of LDPE promoted the decomposition of cotton stalks. Similarly, Fan et al. found that the presence of LDPE could facilitate the decomposition of biomass components through promoting the dehydration and depolymerization of cellulose to produce H_2_O and CO_2_ [23]. Compared with LDPE (474.6 °C), the second peak temperature of the blended samples shifted to the higher temperature range (476.8 °C, 479.8 °C, and 480.5 °C for C3P1, C2P1 and C1P1, respectively, as seen in Table S1), indicating that the presence of cotton stalks could inhibit the decomposition of LDPE. This result is consistent with the study of Kumagai et al., who concluded that PE decomposition into lower-molecular-weight compounds is interrupted by hydrogen donation from beech wood-derived biochar [24].

### 3.2. Biochar Characteristic

#### 3.2.1. The Physicochemical Properties

The physicochemical properties of the biochar are shown in Table 1. The yield of CS-derived biochar slightly decreased from 34.39% to 31.70% with the increase in the pyrolysis temperature because the cotton stalks almost completely decomposed before 400 °C (see Figure 1); however, the pH value and ash content of the biochars showed an opposite trend. The C content in C1P0500 was the highest, accounting for 68.81% of the total weight, while the O and H contents were the lowest (14.13% and 3.17%). In contrast, the changes in N contents were relatively small (1.06–1.13%). The H/C and O/C contents decreased with increasing temperature, suggesting that enhanced aromaticity and weakened polarity occurred at higher temperatures [25,26].

The LDPE addition obviously increased the yield of biochars when pyrolyzed at 400 °C, and the more LDPE was added, the higher the yield of biochar was. However, an opposite result occurred at 450 °C and 500 °C, which was consistent with the result of the TG analysis. Although a slight change in pH was observed after adding the LDPE, this difference was not significant. At 400 °C and 450 °C, the addition of the LDPE significantly increased the C and H contents but decreased the N and O contents, resulting from the incomplete decomposition of LDPE containing higher C and H contents in LDPE than those in cotton stalks. Therefore, a higher addition ratio of LDPE led to higher C and H contents and lower N and O contents. Meanwhile, higher H/C and lower O/C contents were found in these blended biochars, indicating that the structure of the biochar was less stable and polarized. Due to the almost complete decomposition of LDPE at 500 °C, no significant difference in the elemental contents H/C and O/C was found between biochars that were pyrolyzed at 500 °C.

#### 3.2.2. Surface Functional Groups

**Figure 2:**
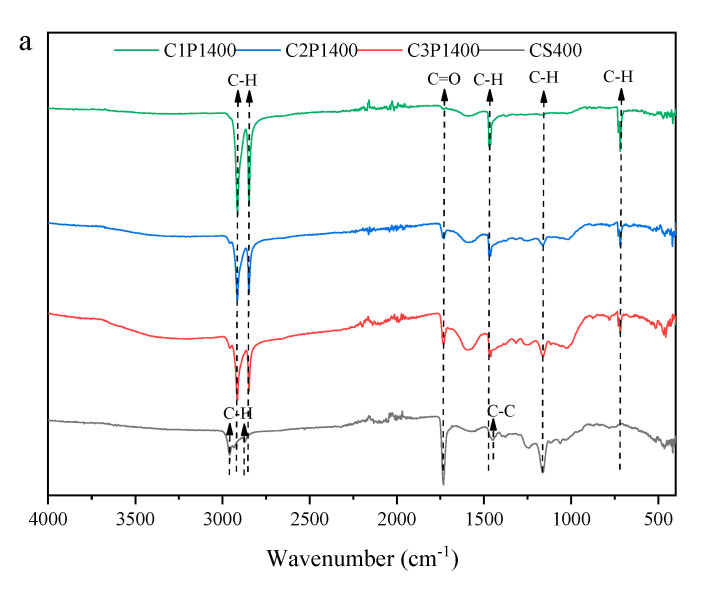

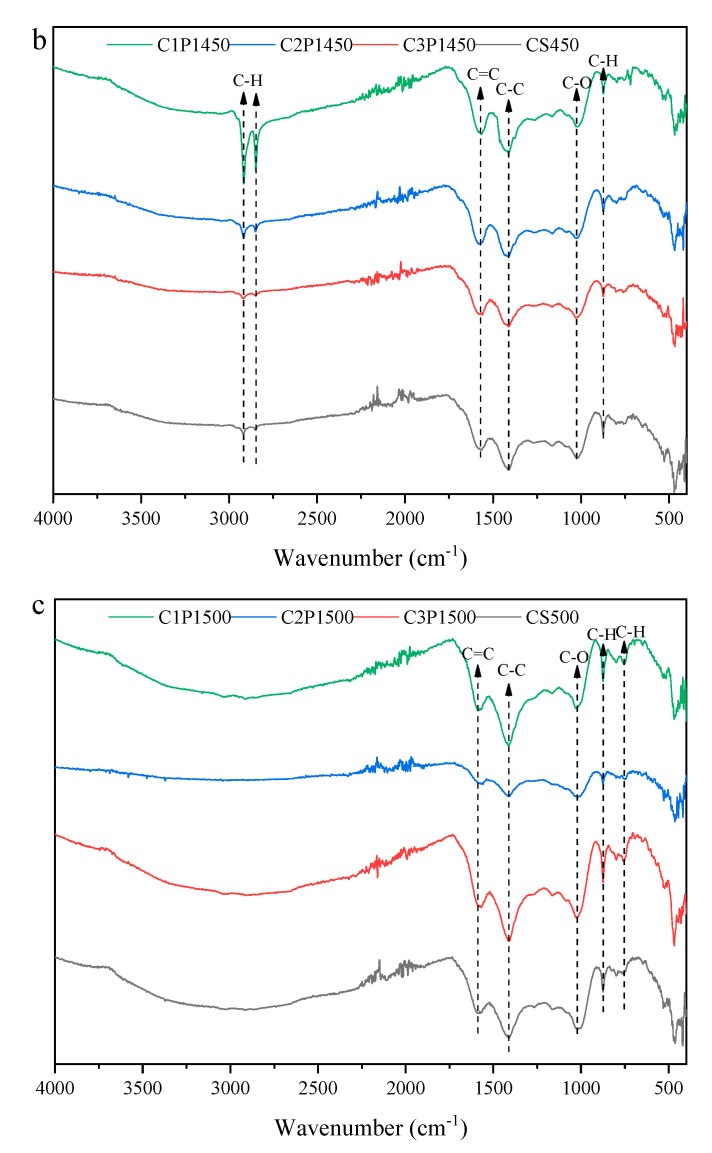
FTIR spectra of biochar pyrolyzed at 400 °C (**a**), 450 °C (**b**), and 500 °C (**c**).

FTIR spectra of biochars are presented in Figure 2. The peaks observed at 1710 cm^−1^ and 1163 cm^−1^ were related to C=O and –OH groups, which was only observed in CS400. The introduction of LDPE decreased the intensity of these two peaks, but led to the appearance of peaks at 2914, 2847, 1470 and 720 cm^−1^, which indicated the stretching vibration of CH_3_ and CH_2_, respectively [27]. It indicated that LDPE had hardly decomposed at 400 °C. When the pyrolysis temperature rose to 450 °C, the C=O and –OH groups disappeared and new bands appeared at 1568, 1412, 1017 and 874 cm^−1^ arising from the stretching vibrations of aromatic C=C, C–C, C–O and C–H bonds [5], suggesting the occurrence of dehydroxylation. Only the bands at 2914 and 2847 cm^−1^ were remarkably enhanced by the addition of LDPE at 450 °C, which resulted from the partial decomposition of LDPE. No noticeable changes in the functional groups were observed between CS500 and the blended biochars. It was confirmed that the interaction between cotton stalks and LDPE occurred through a single physical process rather than chemical reactions.

#### 3.2.3. The Surface Area, Pore Volume, Pore Size of the Biochars

The pore structure variations were characterized by the specific surface area (S_BET_), total pore volume (V_Tot_), and median pore width (MPD) (Table 2). Table 2 shows that the S_BET_ and pore volumes of biochars increased significantly with the increase in the pyrolysis temperature, principally attributed to the escape of volatiles during the thermal decomposition of the organic matter. The C1P0500 had the largest values for S_BET_ and V_Tot_ (9.62 m^2^/g and 0.01182 cm^3^/g, respectively). The MPD of all biochars was below 2 nm, and the ratio of S_Micro_ to S_BET_ and V_Micro_ to V_Tot_ exceeded 0.5, indicating that the microporous type dominated the pore structure types, which was conducive to the adsorption of heavy metal cations [28].

After pyrolysis at 400 °C, the addition of LDPE decreased the S_BET_ and PV. This is because the pores and channels were blocked due to the melted LDPE, as proven by the results of the SEM. However, the SBET, PV, and MPD of the blended biochar increased when the temperature rose to 450 °C and 500 °C. During the thermal decomposition of LDPE, many volatiles such as H_2_ and hydrocarbon were produced [7], thus promoting the formation of new pores and channels in the biochar. Similar results were acquired by Chen et al. (2015) [29], who found that the introduction of high-density polyethylene (HDPE) led to higher BET surface areas and pore volumes when compared to biochars derived from waste newspaper [29].

#### 3.2.4. SEM

**Figure 3:**
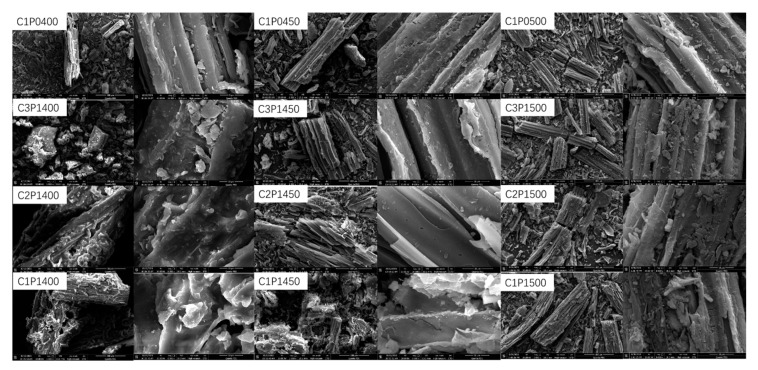
SEM images of biochars.

As shown in Figure 3, C1P0400 showed a smooth surface without visible pores. When the temperature was increased to 450 °C, the control contacts of the biochars became thin and twisted, and the surface of the biochar displayed some macropores. When the pyrolysis temperature was 500 °C, these macropores on the biochar surface vanished gradually owing to its collapse. These results were also confirmed by the aforementioned surface area and porosity data (Table 2).

After the pyrolysis of the mixture at 400 °C, it was seen clearly that the melted LDPE covered the surface of the biochars, which helped to maintain the stability of the overall structure. When the pyrolysis temperature reached 450 °C, the coverage of the LDPE could not be observed due to its almost complete decomposition, as seen in Figure 1. The addition of a large amount of LDPE led to the collapse of the overall structure, and the surface became coarse. More obvious collapses occurred at 500 °C, resulting from the release of many volatiles during the decomposition of LDPE.

### 3.3. Adsorption of Pb(II) on Biochar

#### 3.3.1. Influence of Blending Ratio and Pyrolysis Temperature

**Figure 4:**
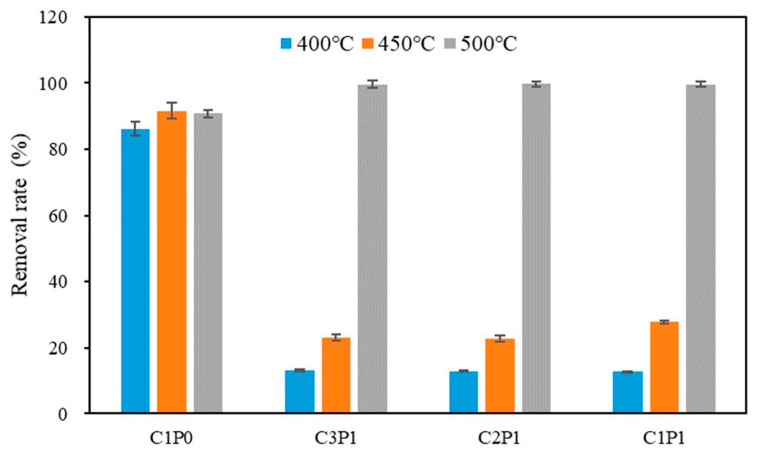
Effect of pyrolysis temperature and mix ratio on the removal efficiency of Pb(II).

The rates of the removal of Pb(II) by all biochars are shown in Figure 4. The biochars obtained at 500 °C exhibited a slightly higher removal ability of Pb(II) in comparison to the biochars pyrolyzed at 400 °C. Kwak et al. (2019) [30] determined that there are relationships among feedstock type, pyrolysis condition, biochar property, and Pb(II) adsorption capacity and found that an increase in pyrolysis temperature increased the Pb(II) adsorption capacity due to the higher pH, higher ash contents, and larger surface areas of biochars by increasing the ion exchange capacity, precipitation, and inner-sphere complexation [30]. Furthermore, Zhang et al. (2020) [31] reported that the surface functional groups such as –OH, C=O, –COO–, and C–O led to more effective Pb(II) removal from aqueous solutions by cow manure-derived biochar [31]. Therefore, there is a slight increase in Pb(II) with increasing temperature, which is attributed to the integrated effect of higher specific surface area and pH and fewer functional groups at higher temperatures.

Compared to the biochar without LDPE, the removal rate of Pb(II) onto the blended biochars that were pyrolyzed at 400 °C and 450 °C substantially decreased, which resulted from the decrease in the adsorption site and the surface area, as proven by the FTIR and BET analysis, respectively. In contrast, an opposite result occurred in the blended biochar pyrolyzed at 500 °C. This was mainly due to a dramatic increase in the surface area. In addition, no significant change in the functional groups occurred between C1P0500 and the blended biochars (Figure 2). These results confirmed that a surface-area-dependent adsorption mechanism could explain the Pb(II) removal by the blended biochar pyrolyzed at 500 °C.

#### 3.3.2. Influence of pH

**Figure 5:**
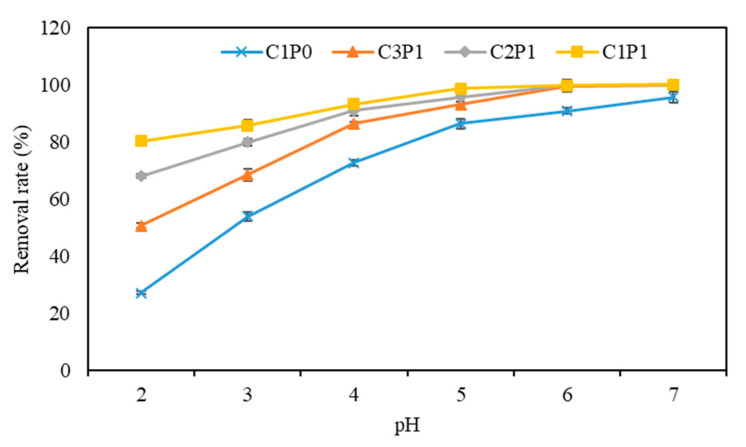
Effect of initial solution pH on Pb(II) removal.

The above results showed that biochars pyrolyzed at 500 °C had the highest removal rate of Pb(II); thus, they were selected for the following experiment. As shown in Figure 5, the removal rate of Pb(II) in all biochars significantly increased with a pH ranging from 2 to 6, which agrees with previous studies [32,33]. Soluble Pb^2+^, PbNO_3_^+^, Pb(NO_3_)_2_, and PbOH^+^ could exist in a solution with a pH < 7.0 [34]. Previous studies have shown that with the increase in the pH, the zeta potentials of biochar became more negative, which was favorable for increasing the removal of the positively charged Pb [35,36]. Thus, chemical reactions might be the dominant process for the adsorption of Pb(II) onto these biochars. It was noted that the removal rate of Pb(II) followed the order of C1P1500 > C2P1500 > C3P1500 > C1P0500, especially at a low-pH range (2–5), and the difference in Pb(II) removal capacity among the samples became larger with a lower pH, indicating that physical adsorption became more dominant during the adsorption process of Pb(II) onto the blended biochar in low-pH conditions. 

#### 3.3.3. Influence of Contact Time

**Figure 6:**
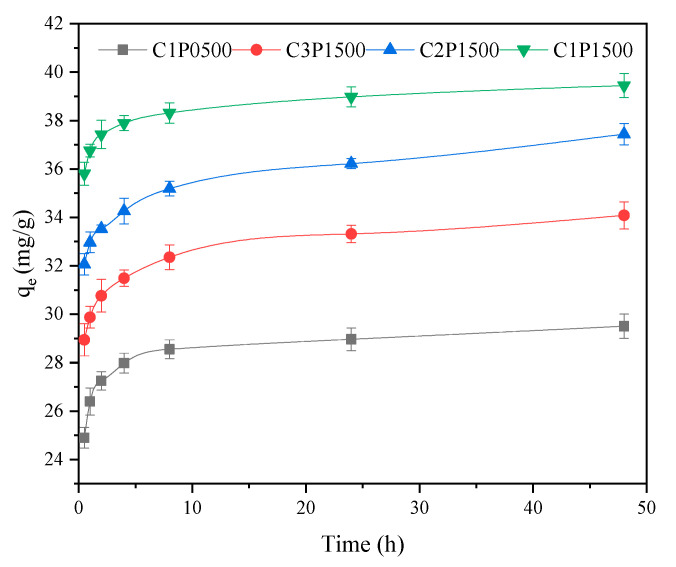
Kinetics of Pb(II) adsorption on biochars pyrolyzed at 500 °C.

The adsorption kinetics of Pb(II) on the C1P0500, C3P1500, C2P1500, and C1P1500 were measured as a function of time (Figure 6). As shown in Figure 6, the adsorption of Pb(II) on all biochars showed two phases: a rapid initial sorption during the first 4 h due to the rapid occupation of easily accessible external sorption sites and a much slower phase that continued until reaching the adsorption equilibrium after about 8 h due to the formation of inner layer complexes or the inhibition of Pb(II) movement through narrow pore channels [37]. As expected, the adsorption capacity of Pb(II) by C1P1500 was the highest at any point during the adsorption process when compared to other biochars. As shown in Table 3, the pseudo-second-order model consistently provided the largest coefficient of determination (R^2^ = 0.975, Table 3) value and showed the best fit to the kinetic adsorption data of C1P0500, suggesting that the adsorption of Pb(II) by C1P0500 was dominated by chemical adsorption [35]. Previous studies have found that Pb(II) adsorption onto sludge biochar [38], corn stalk biochar [39], and pig and cow manure biochars [40] from water follows the pseudo-second-order model. However, the kinetic adsorption of Pb(II) on the blended biochars were better suited to the Elovich kinetic model than other models, with R^2^ values of 0.995, 0.994, and 0.987 for C3P1500, C2P1500, and C1P1500, respectively, indicating that Pb(II) was adsorbed on the highly heterogeneous surfaces of these biochars [41]. The highly heterogeneous surfaces of blended biochars resulted from the collapse of the structure, as shown in Figure 3.

#### 3.3.4. Influence of Initial Concentration

**Figure 7:**
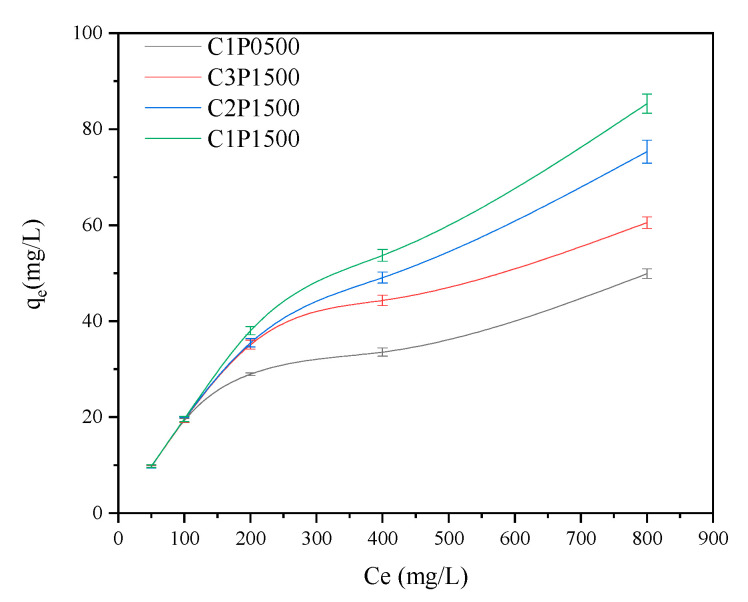
Adsorption isotherms of Pb(II) on biochars pyrolyzed 500 °C.

The adsorption isotherms of Pb(II) at different initial concentrations (50–800 mg L^−1^) are shown in Figure 7. The adsorption capacities of Pb(II) by biochars increased with increasing initial Pb(II) concentration, and they did not achieve equilibrium until 800 mg L^−1^, indicating that no biochar could remove the total amount of Pb(II) from the solution. Table 4 presents the adsorption constants and correlation coefficients of C1P0500, C3P1500, C2P1500, and C1P1500 for Pb(II) from the Langmuir and Freundlich isotherms. The correlation coefficient of the Langmuir model (R^2^ =0.990-0.996, Table 4) of blended biochar was greater than that of the Freundlich model (R^2^ = 0.693–0.851), suggesting that the adsorption of Pb(II) on these biochars were dominated by monolayer chemical adsorption [41]. However, in the case of C1P0500, either the Langmuir or Freundlich models (both have R^2^ values exceeding 0.97) are appropriate for modeling the equilibrium adsorption of Pb(II), indicating that the adsorption of Pb(II) onto C1P0 surfaces were probably energetically homogeneous [36]. The maximum adsorption capacity (q_max_) of C3P1500, C2P1500, and C1P1500 for Pb(II) (89.21, 120.64, and 199.82 mg g^−1^, respectively) was higher than that of C1P0500 (63.05 mg g^−1^), probably because of the increased specific surface area (Table 2). In addition, it is also worth mentioning that the q_max_ of Pb(II) adsorption on C1P1 was much higher than that of other biochars derived from other biomass reported in previous literature (Table S2), suggesting that LDPE-doping biochar can serve as a novel agent for Pb(II) remediation.

## 4. Conclusions

This study investigated the improvement in the characteristics of biochars pretreated from different ratios of cotton stalks and LDPE and their potential ability to adsorb Pb(II) from solutions. A TG analysis revealed that the addition of LDPE promoted the carbonization of cotton stalks. When the pyrolysis temperature was 500 °C, the added LDPE improved the porous properties of the biochars derived from cotton stalks, i.e., it increased the specific surface area and pore volume. The Pb(II) adsorption capacity was mainly dependent on the mix ratios and pyrolysis temperature. Due to its developed pore structure, C1P1500 showed the highest Pb(II) adsorption capacity (199.82 mg g^−1^) in an aqueous solution. Monolayer chemical activity accounted for the Pb(II) adsorption on the highly heterogeneous surface of the blended biochars, as proven by higher correlation coefficients using the Elovich and Langmuir models. These results provide an effective method for treating a mixture of cotton stalks and plastic film as a functional carbon precursor for the adsorption of heavy metals.

## Figures and Tables

**Table 1:** The physicochemical properties of feedstock and biochar.

	pH	Yield (%)	Ash (%)	C (%)	H (%)	N (%)	O (%)	H/C	O/C
Cotton Stalks	7.82 ± 0.08	-	6.21 ± 0.15	42.68 ± 0.33	4.89 ± 0.06	1.69 ± 0.03	44.53 ± 0.16	1.37 ± 0.05	0.696 ± 0.005
LDPE	7.98 ± 0.07	-	-	85.70 ± 0.32	14.30 ± 0.12	-	-	2.00 ± 0.02	-
CSB400	8.43 ± 0.06	34.39 ± 0.24	12.50 ± 0.19	65.75 ± 0.31	3.86 ± 0.01	1.06 ± 0.02	16.83 ± 0.22	0.70 ± 0.01	0.171 ± 0.003
C3P1400	7.99 ± 0.06	49.61 ± 0.35	6.21 ± 0.14	74.70 ± 0.49	8.09 ± 0.02	0.62 ± 0.02	10.38 ± 0.19	1.30 ± 0.02	0.093 ± 0.002
C2P1400	8.08 ± 0.05	54.54 ± 0.29	4.87 ± 0.09	76.69 ± 0.66	9.43 ± 0.06	0.53 ± 0.03	8.49 ± 0.32	1.40 ± 0.03	0.074 ± 0.002
C1P1400	8.26 ± 0.11	65.80 ± 0.22	3.40 ± 0.11	79.92 ± 0.35	11.30 ± 0.05	0.35 ± 0.02	5.03 ± 0.26	1.70 ± 0.04	0.042 ± 0.001
CSB450	9.26 ± 0.09	32.75 ± 0.39	12.73 ± 0.24	68.10 ± 0.38	3.61 ± 0.03	1.06 ± 0.04	14.50 ± 0.33	0.64 ± 0.00	0.142 ± 0.002
C3P1450	9.64 ± 0.08	27.84 ± 0.26	10.99 ± 0.34	69.41 ± 0.39	3.85 ± 0.02	1.02 ± 0.02	14.73 ± 0.28	0.67 ± 0.01	0.141 ± 0.002
C2P1450	9.67 ± 0.08	22.33 ± 0.33	10.48 ± 0.42	69.88 ± 0.11	4.03 ± 0.05	1.04 ± 0.01	15.38 ± 0.23	0.70 ± 0.02	0.148 ± 0.003
C1P1450	9.24 ± 0.11	19.91 ± 0.35	8.60 ± 0.27	71.42 ± 0.42	5.38 ± 0.04	0.96 ± 0.02	13.63 ± 0.15	0.90 ± 0.03	0.127 ± 0.001
C1P0500	10.04 ± 0.12	31.70 ± 0.18	11.96 ± 0.41	68.81 ± 0.38	3.17 ± 0.02	1.13 ± 0.03	14.13 ± 0.17	0.55 ± 0.01	0.137 ± 0.002
C3P1500	10.15 ± 0.10	24.31 ± 0.20	11.11 ± 0.37	69.74 ± 0.33	3.09 ± 0.04	1.13 ± 0.03	14.93 ± 0.25	0.53 ± 0.00	0.143 ± 0.003
C2P1500	9.96 ± 0.11	20.93 ± 0.18	10.42 ± 0.28	70.24 ± 0.27	3.14 ± 0.05	1.06 ± 0.04	15.14 ± 0.19	0.54 ± 0.01	0.144 ± 0.005
C1P1500	10.14 ± 0.09	16.15 ± 0.23	10.34 ± 0.42	70.91 ± 0.36	3.24 ± 0.03	1.04 ± 0.03	14.47 ± 0.12	0.55 ± 0.01	0.136 ± 0.003

**Table 2:** Surface area and pore volume values of biochar.

	S_BET_ (m^2^/g)	S_Micro_ (m^2^/g)	V_Tot_ (10^−2^ cm^3^/g)	V_Micro_ (10^−2^ cm^3^/g)	MPD (Å)
C1P0400	0.83	0.71	0.30	0.24	10.80
C3P1400	0.27	0.16	0.22	0.19	21.48
C2P1400	-	-	-	-	-
C1P1400	-	-	-	-	-
C1P0450	1.24	1.12	0.44	0.42	10.25
C3P1450	2.68	2.09	0.61	0.56	9.64
C2P1450	5.09	3.18	0.42	0.38	15.42
C1P1450	8.49	5.42	1.24	1.22	14.61
C1P0500	9.620	5.846	1.18	1.08	14.89
C3P1500	17.181	11.55	1.27	1.35	15.01
C2P1500	43.966	27.30	3.91	3.84	18.27
C1P1500	68.260	43.51	5.58	5.02	18.04

**Table 3:** Parameters of kinetics models for Pb(II) adsorption on biochars.

	Pseudo-First Order			Pseudo-Second Order			Elovich		
	Qe (mg g^−1^)	K_1_ (h^−1^)	R^2^	Qe (mg g^−1^)	K_2_ (mg (g h)^−1^)	R^2^	a (mg g^−1^)	b	R^2^
C1P0500	28.19	4.17	0.693	28.86	0.40	0.975	26.26	0.95	0.915
C3P1500	32.18	4.06	0.375	32.93	0.34	0.792	29.91	1.09	0.995
C2P1500	34.63	5.01	0.172	35.71	0.32	0.693	32.79	1.11	0.994
C1P1500	37.93	5.37	0.385	38.67	0.55	0.851	36.72	0.74	0.987

**Table 4:** Constants of the isotherm equation for Pb(II) adsorption on biochars.

	Freundlich			Langmuir		
	K_F_	n	R^2^	K_L_	q_max_	R^2^
C1P0500	1.48	0.54	0.975	0.004	63.05	0.981
C3P1500	0.99	0.63	0.792	0.003	89.21	0.990
C2P1500	0.73	0.71	0.693	0.002	120.64	0.992
C1P1500	0.57	0.76	0.851	0.001	199.82	0.996

## Data Availability

The data presented in this work are available in the article and supplementary materials.

## Supplementary Materials

The following supporting information can be downloaded at https://www.mdpi.com/article/10.3390/molecules27154868/s1: Text S1. The analysis of adsorption kinetic and isotherm of Pb(II) on biochar. Table S1. The TG-DTG characteristic parameters of cotton stalk, LDPE and their mixture. Table S2. The maximum Pb((II) adsorption capacities of biochar co-pyrolyzed by cotton stalk and LDPE, other biomass. Figure S1. The experimental setup of vertical pyrolysis furnace. See [42,43,44,45,46,47].

## Sample Availability

Samples of the compounds are not available from the authors.
