# Supplementary material for: Co-Pyrolysis of Cotton Stalks and Low-Density Polyethylene to Synthesize Biochar and Its Application in Pb(II) Removal
Source: Molecules. 2022 Jul 29;27(15):4868. doi: 10.3390/molecules27154868 (PMC9369942; doi:10.3390/molecules27154868)
Supplement: Supplementary file 1 [file molecules-27-04868-s001.zip › molecules-1760666-supplementary.pdf]

Text S1 The analysis of adsorption kinetic and isotherm of Pb(II) on biochar

Pseudo-first order (Eq.1), pseudo-second order (Eq.2), and Elovich (Eq.3) models were used to fit the data of adsorption kinetic, which are expressed as followed:

$$Q_t = Q_e(1-e^{-k_1 t}) \quad (1)$$

$$Qt = (Q_e^2 k_2 t)/(1+Q_e k_2 t) \quad (2)$$

where $Q_t$ (mg $g^{-1}$) and $Q_e$ (mg $g^{-1}$) are the amounts of Pb(II) adsorbed at time t and at equilibrium, respectively, $k_1$ ($h^{-1}$) represents the rate constant of pseudo-first-order model, and $k_2$ is the rate constant of the pseudo second-order model (g $mg^{-1}$ $h^{-1}$).

$$Qt = a + b\ln(t) \quad (3)$$

where $Q_t$ (mg $g^{-1}$) is the amounts of Pb(II) adsorbed at time t, a, and b are constants.

The Langmuir (Eq. 4) and Freundlich (Eq. 5) model were appled to simulate the adsorption isotherm data.

$$Q_e = K_L q_{max} C_e/(1+ K_L C_e) \quad (4)$$

where $Q_e$ (mg $g^{-1}$) and $C_e$ (mg $L^{-1}$) are the adsorbed amount of Pb(II) by biochar and Pb(II) concentration at equilibrium solution, $K_L$ (L $mg^{-1}$) is the Langmuir affinity constant, and $q_{max}$ (mg $g^{-1}$) is the theoretical maximum adsorption capacity as the monolayer surface is completely covered.

$$Q_e = K_F C_e^n \quad (5)$$

where $K_F$ [(mg $g^{-1}$) (L $mg^{-1}$)$^{1/n}$] and n are the constants related to adsorption capacity and intensity, respectively.

**Table S1.** The TG-DTG characteristic parameters of cotton stalk, LDPE and their mixture.

| | First Stage | | | | Second Stage | | | |
|---|---|---|---|---|---|---|---|---|
| | $T_i$ | $T_f$ | $T_{max}$ | Residue at $T_{max}$ (%) | $T_i$ | $T_f$ | $T_{max}$ | Residue at $T_{max}$ (%) |
| CS | 190.2 | 382.6 | 348.8 | 50.19 | - | - | - | - |
| LDPE | - | - | - | - | 390.5 | 503.9 | 474.6 | 35.06 |
| C3P1 | 189.9 | 382.1 | 347.2 | 64.06 | 433.7 | 505.3 | 476.8 | 34.60 |
| C2P1 | 189.5 | 382.4 | 345.7 | 67.97 | 431.5 | 505.0 | 479.8 | 34.35 |
| C1P1 | 189.3 | 381.8 | 344.3 | 76.27 | 425.9 | 504.5 | 480.5 | 34.59 |

**Table S2.** The maximum Pb((II) adsorption capacities of biochar co-pyrolyzed by cotton stalk and LDPE, other biomass.

| | Adsorption Conditions | $Q_{max}$ (mg/g) | Ref. |
|---|---|---|---|
| Cotton stalk+LDPE | pH = 5.0, t = 24 h, 303K | 199.82 | in this study |
| Rice husk | pH = 5.0, t = 24 h, 298K | 2.40 | [1] |
| Pinewood | pH = 5.0, t = 24 h, 298K | 4.25 | |
| British broadleaf hardwood | pH = 5.0, t = 24 h, 293K | 47.66 | [2] |
| Corn stalk | pH = 5.5, t = 12h, 298K | 49.70 | [3] |
| Rice husk | - | 26.70 | [4] |
| Pine needle | pH = 5.0, t = 48 h , 308K | 40.43 | [5] |
| Saw dust | pH = 5.0, t = 24 h, 298K | 62.68 | [6] |


[1] Z. Liu, F.-S. Zhang, Removal of lead from water using biochars prepared from hydrothermal liquefaction of biomass, Journal of Hazardous Materials, 167 (2009) 933-939.

[2] Z. Shen, F. Jin, F. Wang, O. McMillan, A. Al-Tabbaa, Sorption of lead by Salisbury biochar produced from British broadleaf hardwood, Bioresource Technology, 193 (2015) 553-556.

[3] L. Liu, Y. Huang, S. Zhang, Y. Gong, Y. Su, J. Cao, H. Hu, Adsorption characteristics and mechanism of Pb(II) by agricultural waste-derived biochars produced from a pilot-scale pyrolysis system, Waste Management, 100 (2019) 287-295.

[4] J. Shi, X. Fan, D.C.W. Tsang, F. Wang, Z. Shen, D. Hou, D.S. Alessi, Removal of lead by rice husk biochars produced at different temperatures and implications for their environmental utilizations, Chemosphere, 235 (2019) 825-831.

[5] V. Choudhary, M. Patel, C.U. Pittman, D. Mohan, Batch and Continuous Fixed-Bed Lead Removal Using Himalayan Pine Needle Biochar: Isotherm and Kinetic Studies, ACS Omega, 5 (2020) 16366-16378.

[6] S. Cheng, Y. Liu, B. Xing, X. Qin, C. Zhang, H. Xia, Lead and cadmium clean removal from wastewater by sustainable biochar derived from poplar saw dust, Journal of Cleaner Production, 314 (2021) 128074.

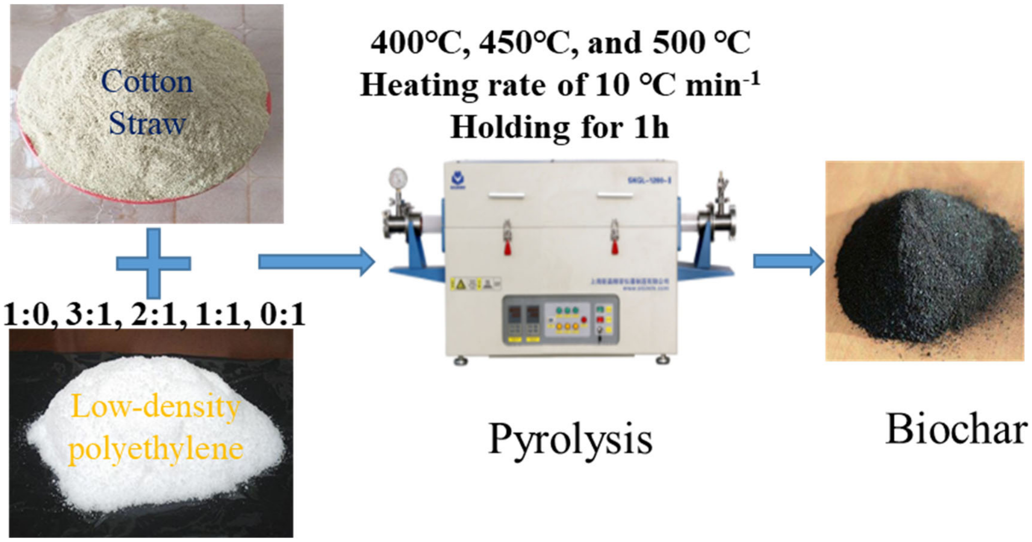


**Figure S1.** The experimental setup of vertical pyrolysis furnace.
